# Influence of Congruency Design on the Contact Stress of a Novel Hinged Knee Prosthesis Using Finite Element Analysis

**DOI:** 10.1111/os.12640

**Published:** 2020-03-11

**Authors:** Jing‐yu Zhang, Dong‐mu Tian, Zhi‐peng Ren, Yong‐cheng Hu, Xiu‐chun Yu

**Affiliations:** ^1^ Department of Bone and Soft Tissue Oncology Tianjin Hospital Tianjin China; ^2^ Department of Hand Surgery, Second Hospital of Tangshan Tangshan China; ^3^ Beijing Weigao Yahua Artificial Joint Development Company Beijing China; ^4^ Department of Orthopaedics The 960th Hospital of the PLA Joint Logistice Support Force Jinan China

**Keywords:** Congruency, Contact area, Contact stress, Finite element analysis, Rotating hinge knee prosthesis

## Abstract

**Objectives:**

To investigate the contact stress and the contact area o tibial inserts and bushings with respect to different congruency designs in a spherical center axis and rotating bearing hinge knee prosthesis under gait cycle loading conditions using finite element analysis.

**Methods:**

Nine prostheses with different congruency (different degrees of tibiofemoral conformity and different distances between the spherical center and the bushing) designs were developed with the same femoral and tibial components. The models were transferred to finite element software. The peak contact stresses and contact areas on tibial inserts and bushings under the gait cycle loading conditions were investigated and compared.

**Results:**

For tibial insert, the peak contact stress was the highest in the low conformity‐long group (61.4486 MPa), and it was 1.88 times higher than that in the group with the lowest stress (moderate conformity‐short group, 32.754 MPa). The contact area was the largest in the low conformity‐long group (420.485 mm^2^), and it was 1.19 times larger than that in the group with the smallest area (moderate conformity‐middle group, 352.332 mm^2^). For bushing, the peak contact stress was the highest in the high conformity‐long group (72.8093 MPa), and it was 3.21 times higher than that in the group with the lowest stress (high conformity‐short group, 22.6928 MPa). The contact area was the largest in the low conformity‐short group (2.41 mm^2^), and it was 2.27 times larger than that in the group with the smallest area (high conformity‐middle group, 1.063 mm^2^).

**Conclusion:**

The results of our study showed that the congruency of the tibiofemoral surface and bushing surface should be considered carefully in the design of the spherical center axis and rotating bearing hinge knee prosthesis. Different levels of contact performance were observed with different congruency designs. In addition, the influence of contact stress and contact area on the polyethylene wear of rotating hinge knee prostheses should be confirmed with additional laboratory tests.

## Introduction

For patients suffering from malignancies around the knee or certain types of gonarthrosis, rotating hinge knee (RHK) arthroplasty is an effective reconstruction method because it yields a satisfactory appearance, early weight‐bearing, and satisfactory restoration of function^1^, ^2^, ^3^. As with all replacement prostheses, the longevity of the hinge knee prosthesis is of concern^4^. Aseptic loosening, infection, and bushing wear are the most common mid‐term to long‐term complications related to prostheses, which always require revision^4^, ^5^, ^6^, ^7^, ^8^. In addition, in contrast to a normal biological joint, most RHK prostheses can only allow biaxial motion and incomplete weight‐bearing by the tibial condyle.

Ultra‐high‐molecular‐weight polyethylene (UHMWPE) wear is a one of the main causes of RHK prosthetic failure, which will lead to bushing damage and osteolysis caused by wear particle disease^4^, ^9^, ^10^. An *in vitro* wear test is a standard test used to evaluate prosthetic wear performance; however, the considerable expense and long duration limit the use of the test^9^. A significant contributor of UHMWPE wear is the contact stress on the UHMWPE surface, which has been investigated to optimize the design of prostheses^11^, ^12^, ^13^. Decreasing contact stress is important for reducing wear and improving prosthetic survival^14^, ^15^. Factors that influence contact stress include the tibiofemoral conformity, the UHMWPE thickness, and the contact area^11^, ^14^.

Bartel *et al.* 16 examined the effect of conformity, thickness, and material on contact stress in UHMWPE for joint prostheses using finite element (FE) analysis, and the authors found that the contact stress on the tibial insert was reduced most when the tibiofemoral surfaces showed more conformity in the medial‐lateral direction and that the thickness of the tibial insert should be maintained to be more than 8 to 10 mm. D'Lima *et al.* 17 evaluated the effect of knee alignment and tibiofemoral conformity on contact stress using FE analysis. The results showed that increasing tibiofemoral conformity reduced contact stress when the knee was well aligned and that malalignment in prosthetic axial rotation was detrimental. Simpson *et al.* 18 also investigated the effect of tibiofemoral conformity and thickness on stress for unicompartmental prostheses using FE analysis during a simulated step‐up activity. The results regarding tibiofemoral conformity were similar to those of the aforementioned studies. Regarding the thicknesses of the tibial insert, a thicker UHMWPE resulted in less contact stress for the noncongruent design; however, the thickness had little effect on the fully congruent design. However, all the aforementioned studies focused on unconstraint prostheses. To the best of our knowledge, none of the studies evaluated the effect of congruency designs on the contact stress of RHK prostheses.

Accordingly, the purposes of this study were: (i) to establish different tibiofemoral conformity designs and different sizes of bushing in FE models of a novel RHK prosthesis; (ii) to investigate the contact stress and the contact area on the tibial insert and bushing during a gait cycle; and (iii) to evaluate the effect of congruency designs on contact stress and contact area among the different FE models.

## Materials and Methods

### *Design Procedure for a Spherical Center Axial Prosthesis*

**Figure 1:**
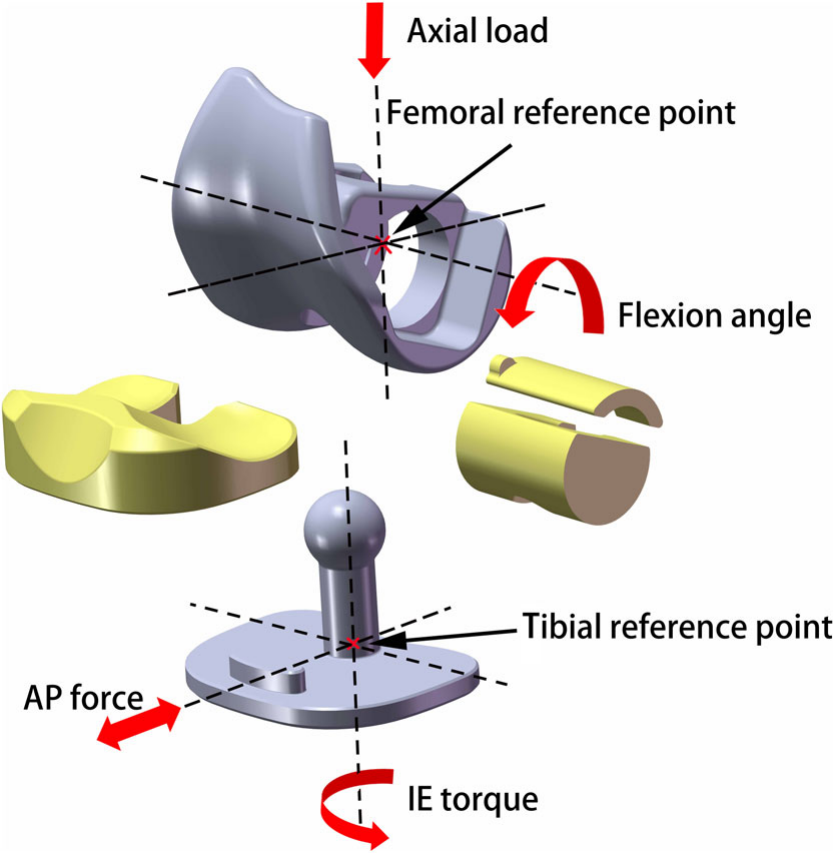
CAD model of the rotating hinge knee prosthesis and loading condition used in this study: the axial force and flexion motion were applied to the femoral reference point, while the AP force and IE torque were applied to the tibial reference point. AP, anterior–posterior; IE, internal–external.

A 3D model (Fig. 1) of the novel RHK prosthesis was developed for this study using Unigraphics NX (Siemens PLM Software) by Wego (Beijing, China). In contrast to the motion axis in the conventional RHK prosthesis, the motion axis (bending axis and rotating axis) of this prosthesis was set as the spherical center axis (SA). In addition, the tibial insert was designed as a rotating bearing (internal/external rotation of 12°).

**Figure 2:**
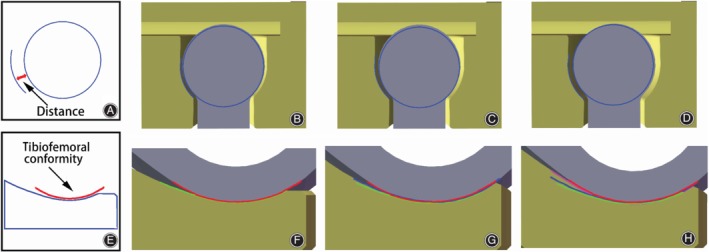
The different congruency designs of the prosthesis: (A) Distance between the spherical center and the bushing; (B) short distance; (C) middle distance; (D) long distance; (E) tibiofemoral conformity; (F) low conformity; (G) middle conformity; and (H) high conformity.

We designed nine prosthetic models with different tibial inserts and different bushings. According to the distance between the spherical center and the bushing (DSB), the bushings were divided into three groups: short 0.5, middle 1.0, and long 1.5 (Fig. 2A–D). In addition, according to the degree of tibiofemoral conformity, the tibial inserts were divided into three groups: low conformity (LC) 60°, moderate conformity (MC) 75°, and high conformity (HC) 90° (Fig. 2E–H). Therefore, we analyzed the peak contact stress on the tibial inserts and bushings of these novel RHK prosthesis with respect to the degree of tibiofemoral conformity and the distance of the bushing. There were nine groups: LC‐short, LC‐middle, LC‐long, MC‐short, MC‐middle, MC‐long, HC‐short, HC‐middle, and HC‐long.

### *Computational Models of the Designs*

Solid modeling and meshing were performed using ABAQUS 6.14–2 (Simulia, Providence, RI). The cobalt chromium molybdenum femoral and tibial components were modeled as rigid bodies. The UHMWPE tibial insert and bushing were modeled as a linear elastoplastic material^19^. Linear tetrahedral elements were used to model the tibial inserts and bushings. The contact condition for the tibiofemoral surface and spherical center‐bushing surface was set as penalty contact with a friction coefficient of 0.04^19^. The convergence test was performed for the appropriate mesh densities in the prosthetic component. The material properties and the element size of the contact surface of the prosthetic component are presented in Table 1.

**Table 1:** Material properties and element size for the finite element model

Component	Young's modulus (MPa)	Poisson's ratio	Element sizes
Femoral component	210 000	0.3	1.2
Tibial component	210 000	0.3	1
Tibial insert	940	0.46	1
Rotation bushing	940	0.46	0.6
Bushing lock	940	0.46	1

### *Finite Element Model Verification*

We performed a biomechanical test to investigate the contact area on the tibiofemoral surface under 3000 N using low‐pressure threshold Fujifilm (LLLW, measuring range 0.2–0.6). The tests were performed at the 0°, 30°, 60°, 90°, and 110° incline positions of the femoral component. The same loading conditions and boundary conditions were applied for FE analysis. The results of the biomechanical test and FE analysis were compared to validate the FE model.

### *Simulation During a Gait Cycle*

**Figure 3:**
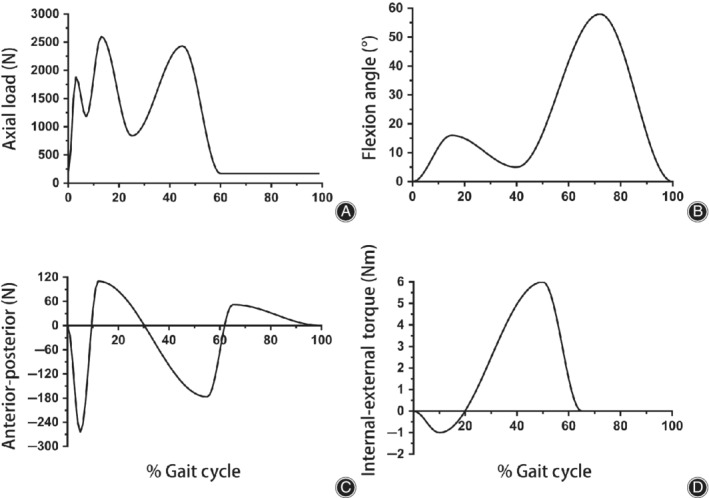
The loading conditions for finite element model based on the ISO gait cycle: (A) Axial load; (B) flexion angle; (C) anterior–posterior force; and (D) internal‐external torque.

We evaluated the peak contact stress on the tibial inserts and bushings of the prostheses in the nine different groups. The loading conditions and kinematic conditions in the FE simulation were adopted from the ISO 14243‐1 standard: maximum load of 2600 N, flexion angle of 0° to 58°, anterior–posterior (AP) force of −265 N to 110 N, and internal‐external (IE) torque of −1 to 6 Nm (Fig. 3). The axial force and flexion motion were applied to the femoral reference point, which was offset medially by 5 mm from the femoral center to simulate physiological loading, while the AP force and IE torque were applied to the tibial reference point (Fig. 1).

The femoral component was constrained in AP directions, medial–lateral directions and IE rotation directions. It was free to translate in the flexion–extension, inferior–superior directions and varus‐valgus rotation directions. The tibial component was constrained in medial‐lateral, inferior–superior, flexion‐extension, and varus‐valgus rotation directions. It was free to translate in the AP and IE rotation directions.

### *Statistical Analysis*

Measurement data (peak contact stress and contact area) were compared with the Tukey test. All statistical analyses were performed using IBM SPSS Statistics 22.0 and a two‐tailed *P* < 0.05 was considered statistically significant difference.

## Results

### *Finite Element Model Validation*

In the biomechanical test, the largest contact area was 512.8 ± 84.11 mm^2^ at 60° of flexion, and the smallest contact area was 386.25 ± 65.94 mm^2^ at 110° of flexion; in the FE analysis, the largest contact area was 484.97 mm^2^ at 60° of flexion, and the smallest contact area was 354.75 mm^2^ at 110° of flexion. The differences between the biomechanical test and FE analysis are shown in Table 2.

**Table 2:** Contact area (mm^2^) difference between biomechanical test and finite element analysis

Groups	Biomechanical test (BT)			Finite element analysis (FEA)	Difference (%)
0°	357.1	392.5	461.6	383.66	4.97
30°	416.8	441.1	563.1	436.81	7.78
60°	429.5	511.2	597.7	484.97	5.43
90°	358.6	427.7	483.7	388.27	8.28
110°	323.8	379.75	455.2	354.75	8.16

Diff (%) = (BT‐FEA)/BT•100

### *Effects of Congruency on the Peak Contact Stress and Contact Area of the Tibial Insert*

#### *Peak Contact Stresses on Tibial Insert*

**Figure 4:**
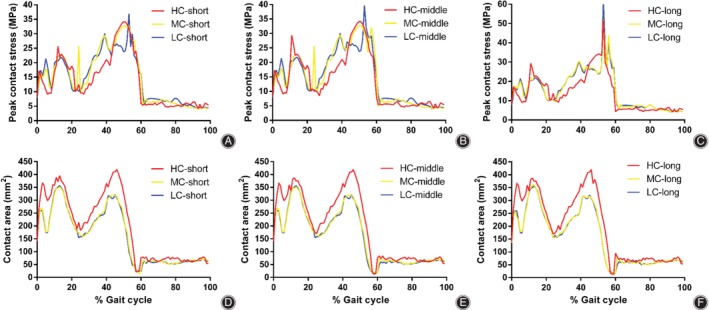
Comparison of the contact characteristics the different congruency tibial insert designs during a gait cycle. (A‐C) Peak contact stress of the short, middle, and long DSB bushing, respectively: the peak contact stresses in the HC groups were slightly lower than those in other groups, followed by MC groups and LC groups; (D‐F) contact area of the short, middle, and long DSB bushing, respectively: the contact areas in the HC groups were slightly larger than those in other groups, followed by MC groups and LC groups. DSB, distance between the spherical center and the bushing; HC, high conformity; MC, middle conformity; LC, low conformity.

**Figure 5:**
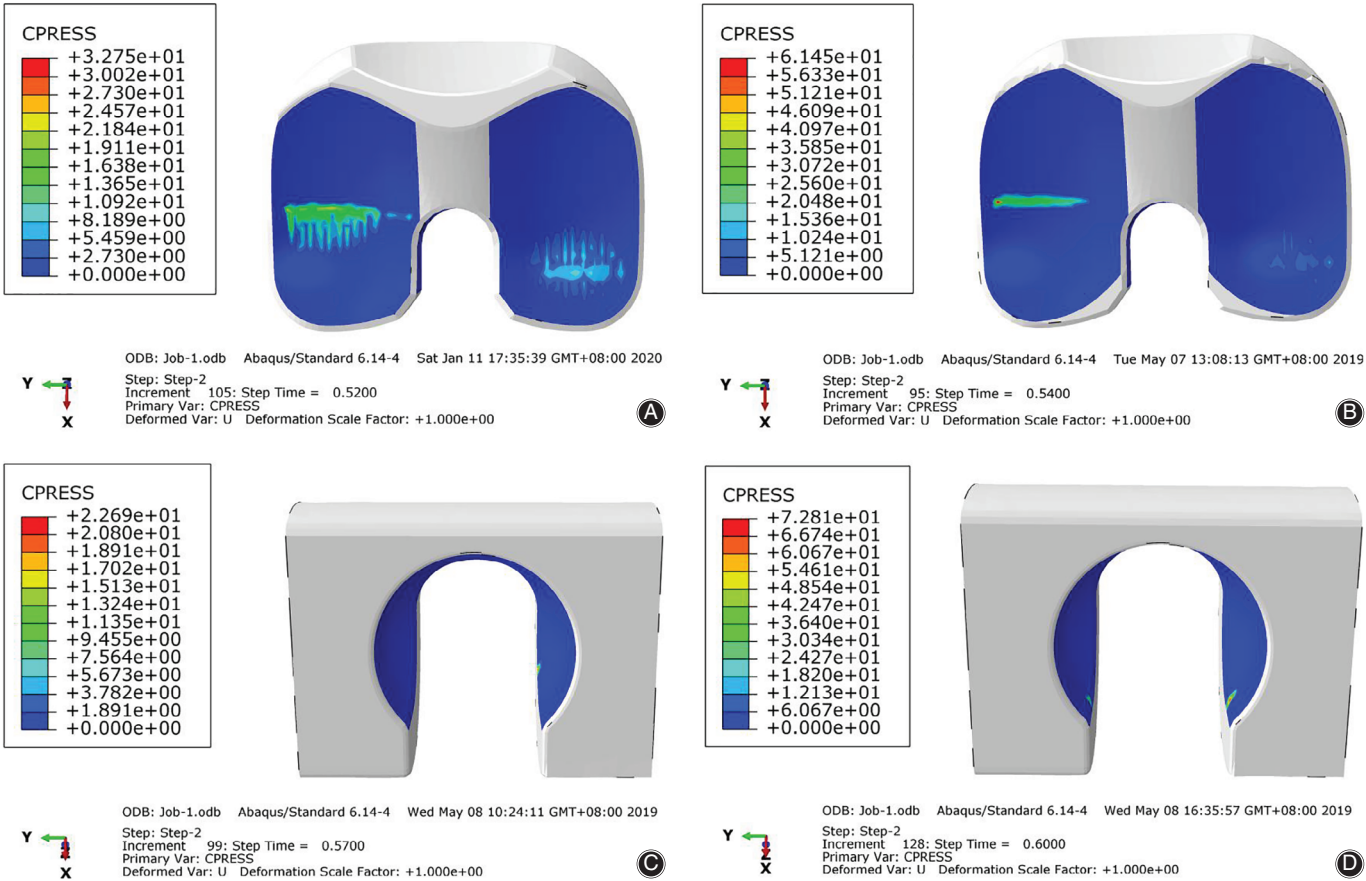
The highest and lowest peak contact stress distribution on the tibial insert and bushing of the prosthesis: (A) The lowest peak contact stress (32.754 MPa) on tibial insert occurred in the middle conformity‐short group; (B) the highest peak contact stress (61.4486 MPa) on tibial insert was occurred in the low conformity‐long group; (C) the lowest peak contact stress (22.6928 MPa) on bushing occurred in the high conformity‐short group; (D) and the highest peak contact stress (72.8093 MPa) on bushing occurred in the high conformity‐long group.

The peak contact stresses on the tibial inserts of different congruency design models during a gait cycle are shown in Fig. 4A–C. The trend of peak contact stress was similar to that of axial force, which increased during the stance phase but decreased during the swing phase. There was no significant difference among the nine groups. The peak contact stress was the highest in the LC‐long group (61.4486 MPa), and it was 1.88 times higher than that in the group with the lowest stress (MC‐short group, 32.754 MPa). In the groups with the same DSB bushing, the contact stresses in the HC groups were slightly lower than those in other groups, followed by MC groups and LC groups. In the groups with the same degree of tibiofemoral conformity, the contact stresses in the short DSB groups were slightly lower than those in other groups, followed by middle DSB groups and long DSB groups. The highest and lowest peak contact stress distributions of the tibial insert are shown in Fig. 5A, B.

#### *Contact Areas on Tibial Insert*

The contact areas on the tibial insert of different congruency design models during a gait cycle are shown in Fig. 4D–F. There was no significant difference among the nine groups. The contact area was the largest in the LC‐long group (420.485 mm^2^), and it was 1.19 times larger than that in the group with the smallest area (MC‐middle group, 352.332 mm^2^). In the groups with the same DSB bushing, the contact areas in the HC groups were slightly larger than those in other groups, followed by MC groups and LC groups. In the groups with the same degree of tibiofemoral conformity, the contact areas were very similar among the different DSB bushing groups.

### *Effects of Congruency on the Peak Contact Stress and Contact Area of the Bushing*

#### *Peak Contact Stresses on Bushing*

**Figure 6:**
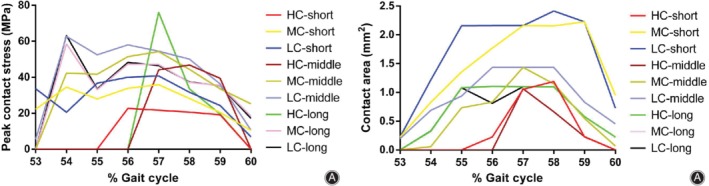
Comparison of the contact characteristics for the different congruency bushing designs during a gait cycle. (A) Peak contact stress: In the groups with the same DSB bushing, the peak contact stresses in the HC groups were lower than those in other groups, while with the same degree of tibiofemoral conformity, the peak contact stresses in the short DSB groups were lower than those in other groups. (B) Contact area: In the groups with the same DSB bushing, the contact areas in the HC groups were lower than those in other groups, while with the same degree of tibiofemoral conformity the contact areas in the middle DSB groups were lower than those in other groups. DSB, distance between the spherical center and the bushing; HC, high conformity; LC, low conformity; MC, middle conformity.

The peak contact stresses on the bushings of different congruency design models during a gait cycle are shown in Fig. 6. In contrast to the contact stress on tibial insert, the contact stress on bushings only occurred during 53%‐60% of the gait cycle, when the ratios of rotation torque/axial force were higher than 1. There was no significant difference among the nine groups. The peak contact stress was the highest in the HC‐long group (72.8093 MPa), and it was 3.21 times higher than that in the group with the lowest stress (HC‐short group, 22.6928 MPa). In the groups with the same DSB bushing, the contact stresses in the HC groups were lower than those in other groups, followed by MC groups and LC groups. In the groups with the same degree of tibiofemoral conformity, the contact stresses in the short DSB groups were lower than those in other groups, followed by long DSB groups and middle DSB groups. The highest and lowest peak contact stress distributions of the bushing are shown in Fig. 5C, D.

#### *Contact Areas on Bushing*

The contact area on the bushing of different congruency design models during a gait cycle are shown in Fig. 6. There was no significant difference among the nine groups. The contact area was the largest in the LC‐short group (2.41 mm^2^), and it was 2.27 times larger than that in the group with the smallest area (HC‐middle group, 1.063 mm^2^). In the groups with the same DSB bushing, the contact areas in the HC groups were lower than those in other groups, followed by MC groups and LC groups. In the groups with the same degree of tibiofemoral conformity, the contact areas in the middle DSB groups were lower than those in other groups, followed by short DSB groups and long DSB groups.

## Discussion

The most important finding in this study was that the congruency design of the novel RHK prosthesis truly influences the contact stress and contact area of the tibial insert and bushing. The parameters of contact stress and contact area are important for optimizing prosthesis designs because they are related to UHMWPE wear, which is the main cause of knee prostheses failure^14^. In addition, our approach and results on the bushings of RHK prostheses can be used as a reference for future studies.

In our study, the peak contact stress on the tibial insert and bushing increased as the degree of tibiofemoral conformity decreased, but with the DSB increased. However, the contact area on the tibial insert increased as the degree of tibiofemoral conformity increased, and no relationship was found between the area and DSB; for the bushing, the contact area increased as the degree of tibiofemoral conformity decreased, but with DSB increased. D'Lima *et al* 17 reported that repeated cyclic loading at yield stress levels ranging from 13 to 32 MPa results in UHMWPE structural failure. For the tibial insert, the long DSB groups had a peak contact stress that was higher than 32 MPa, but for the bushing, only the HC‐short group had a peak contact stress lower than 32 MPa. Previous studies^20^ showed that the contact areas of most total knee prostheses range from 80 to 300 mm^2^. For the tibial insert, the contact areas range from 352.332 to 420.485 mm^2^, indicating that our prosthesis had a high conformity design, which provided a large contact area.

The most common types of mechanical failures of RHK prostheses are aseptic loosening and bushing wear^4^, ^5^, ^7^. Aseptic loosening is also related to UHMWPE wear, which leads to wear particle disease, resulting in osteolysis^9^. Wimmer *et al.* 21 performed a retrieval study and reported that: adhesion and abrasion wear (polishing) are determined by the sliding distance and shear force; surface fatigue wear (pitting and delamination) is determined by contact stress; and abrasive wear is caused by third bodies. Therefore, increased tibiofemoral conformity can decrease the sliding distance/shear force and contact stress, finally decreasing the amount of adhesion/abrasion wear and surface fatigue wear^16^, ^22^, ^23^, ^24^, ^25^. Yang and Lin performed FE analysis of the contact characteristics on a novel RHK prosthesis and reported that a design with high conformity can increase the contact area and decrease the contact stress, finally decelerating the early wear^14^.

However, different opinions on the relationship between contact characteristics and wear have been reported. Galvin *et al.* 26 performed a wear test on different conformity designs and found that lower conformity prostheses provided higher contact stresses and smaller contact areas but lower levels of UHMWPE wear. The same results were found by Brockett *et al*., who performed a wear test on prostheses with two different degrees of conformity^27^. The authors thought that the higher degree of tibiofemoral conformity would trap the third‐body debris within the prosthetic joint, leading to more severe joint surface damage, and that the constraints of the joint would generate secondary stresses to promote fatigue wear^27^. In contrast to the total knee prostheses used in the aforementioned studies, our prosthesis has a highly constrained design that hinders motion and leads to a high contact stress zone. Therefore, more studies on the influence of contact stresses and contact area on the UHMWPE wear of RHK prostheses should be performed in the future.

There are several limitations to our study. First, only the SA prosthesis was investigated. Prostheses from other manufacturers with different motion axes may yield different results. Second, we simulated only a gait cycle, and more simulations, such as those during running, stair ascent, and squatting, should be performed to investigate the contact performance of this novel RHK prosthesis. In addition, only the prosthesis itself was investigated in our study, and the musculoskeletal system should also be investigated to determine the resultant force on the prosthesis, especially the spherical joint.

### *Conclusion*

The results of our study showed that the congruency of the tibiofemoral surface and bushing surface should be considered carefully in the design of the SA and the rotating bearing hinge knee prosthesis. Different levels of contact performance were observed with different congruency designs. In addition, the influence of contact stress and contact area on the UHMWPE wear of RHK prostheses should be confirmed with additional laboratory tests.

## Disclosure

This research did not receive any specific grant from funding agencies in the public, commercial, or not‐for‐profit sectors.
